# Control of occult hepatitis B virus infection

**DOI:** 10.1515/almed-2022-0065

**Published:** 2022-08-15

**Authors:** Marta Lalana Garcés, Oihana Ortiz Pastor, Gemma Solé Enrech, Armando R. Guerra-Ruiz, Gregori Casals Mercadal, Alejandro Almería Lafuente, María Antonieta Ballesteros Vizoso, Pablo Gabriel Medina, Sergio Salgüero Fernández, Angielys Zamora Trillo, Isabel Aured de la Serna, Juan Carlos Hurtado, Sofía Pérez-Del-Pulgar, Xavier Forns, Manuel Morales Ruiz

**Affiliations:** Comisión de Valoración Bioquímica de la Enfermedad Hepática, Sociedad Española de Medicina de Laboratorio (SEQC-ML), Barcelona, Spain; Servicio de Análisis Clínicos, Hospital de Barbastro, Huesca, Spain; Servicio de Bioquímica Clínica, Hospital Universitario Miguel Servet, Zaragoza, Spain; Servei de laboratori, UDIAT-CD, Corporació Sanitaria Parc Taulí, Sabadell, Spain; Servicio de Análisis Clínicos, Hospital Universitario Marqués de Valdecilla, Santander, Spain; Servicio de Bioquímica y Genética Molecular, CDB, Hospital Clínic de Barcelona, IDIBAPS, CIBEREHD, Barcelona, Spain; Servicio de Bioquímica Clínica, Hospital Royo Villanova, Zaragoza, Spain; Servicio de Análisis Clínicos, Hospital Universitario Son Espases, Palma de Mallorca, Spain; Servicio de Bioquímica Clínica, Hospital Universitari Vall d’Hebron, Barcelona, Spain; Servicio de Análisis Clínicos, Hospital Universitario Fundación Alcorcón, Madrid, Spain; Servicio de Bioquímica Clínica, Hospital General Universitario Gregorio Marañón, Madrid, Spain; Servicio de Digestivo, Hospital de Barbastro, Huesca, Spain; Servicio de Microbiología, CDB, Hospital Clínic de Barcelona, Universitat de Barcelona, Barcelona, Spain; Instituto de Salud Global de Barcelona (ISGlobal), Barcelona, Spain; Servicio de Hepatología, Hospital Clínic de Barcelona, IDIBAPS, CIBEREHD, Barcelona, Spain; Departamento de Biomedicina de la Facultad de Medicina y Ciencias de la Salud-Universidad de Barcelona, Barcelona, Spain

**Keywords:** covalently closed circular DNA (cccDNA), occult HBV infection (OBI), ultra-sensitive hepatitis B virus surface antigen (ultra-sensitive HBsAg)

## Abstract

**Background:**

The diagnosis of hepatitis B virus (HBV) infection requires HBV DNA testing and serologic testing for detection of the surface antigen (HBsAg) and the hepatitis B core antibody (anti-HBc). There is a population of patients with occult HBV infection (OBI), which is not detected by HBsAg or HBV DNA quantification in blood, despite the presence of active replication in the liver.

**Scope:**

This document provides a definition of OBI and describes the diagnostic techniques currently used. It also addresses the detection of patients with risk factors and the need for screening for OBI in these patients.

**Summary:**

Correct diagnosis of OBI prevents HBV reactivation and transmission. Diagnosis of OBI is based on the detection of HBV DNA in patients with undetectable HBsAg in blood.

**Perspectives:**

A high number of patients with OBI may remain undiagnosed; therefore, screening for OBI in patients with factor risks is essential. For a correct diagnosis of OBI, it is necessary that new markers such as ultrasensitive HBsAg are incorporated, and a more comprehensive marker study is performed by including markers such as cccDNA.

## Definition of occult hepatitis B virus infection (HBV)

Occult hepatitis B virus (HBV) infection (OBI) is defined as the presence of detectable HBV DNA in the liver and/or serum of surface antigen (HBsAg) negative carriers.

When HBV DNA load is detected, it is generally very low: <200 IU/mL (1,000 copies/mL) [1]. Some studies, however, report even lower viral loads in serum in more than 90% of patients with OBI (20 IU/mL [2]). Other studies reveal that HBV DNA is only intermittently detected in serum/plasma [3]. In addition, liver enzyme markers such as ALT (alaninoaminotransferasa) are generally within normal range in these patients [2].

Based on serologic patterns, OBI is classified into: i) Seropositive OBI: the patient has HBV core antibodies (anti-HBc) and/or positive HBV surface antibodies (anti-HBs); and ii) seronegative OBI: with negative anti-HBc and anti-HBs [1]. All patients present detectable HBV DNA in hepatic tissue (Table 1) [4].

**Table 1:** Markers in serum and the liver of OBI patients.

	Serum		Liver	
	Anti-HBc	Anti-HBs	HBV DNA	HBV DNA
OBI seropositive	+	+	<200 IU/mL/undetectable	+
OBI seronegative	−	−	<200 IU/mL/undetectable	+

OBI, occult hepatitis B virus infection.

In seropositive OBI, HBsAg becomes undetectable some months (or years in the case of chronic infection) after the resolution of acute hepatitis. In immunological terms, it is unknown whether patients with HBV infection or chronic HBV infection who lose HBsAg with antiviral therapy are comparable to those who present spontaneous HBsAg clearance. The potential implications of this difference are unclear [3].

The percentage of seronegative patients ranges from 1 to 22% of patients with OBI [5, 6]. These patients may either have progressively lost HBV antibodies (anti-HBc and anti-HBs) or have been negative since early infection [3].

**Figure 1:**
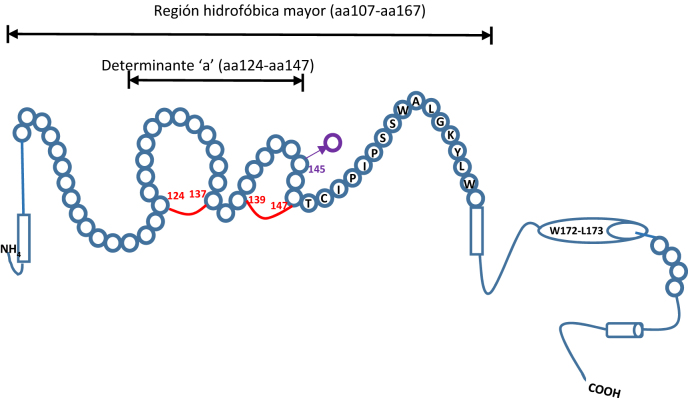
Second HBsAg structure. Major hydrophobic regions (amino acids 107–167) and the “a” determinant (amino acids 124–147) are represented by circles with the name of the respective amino acid (1-letter code). The “a” determinant is the immunodominant epitope, which is maintained through two disulfide bonds *C124–C137* and *C139–C147*. One of the most frequent mutations is shown in violet. Adapted from Rios-Ocampo et al. and Jaramillo et al. [4, 8].

Additionally, there is a subgroup of OBI patients termed as “false OBI”, carriers of mutations in HBsAg, that are not recognized by some routine detection assays. These cases exhibit more elevated HBV DNA levels, resembling those found in HBsAg-positive HBV infection [1, 7]. These patients are infected by variants with a mutation in the S gene, known as “escape variants”. These are mutations in the sequence that encodes the “a” determinant (Figure 1) [4, 8]. The most common and best characterized mutants (glycine-to-arginine substitution at position 145, [G145R]) have been consistently found in the second “a” determinant loop. This mutation was first described by Carman et al. in a newborn to a carrier mother, who was immunized and treated with specific immunoglobulins after birth, but ultimately presented HBV infection [9], [10], [11].

Escape variants pose a significant risk. On the one hand, some HBsAg detection immunoassays may yield false negative results; otherwise said, HBsAg are not recognized by some commercially-available assays. In addition, the vaccines currently used for HBV may not be effective for infection due to the presence of mutants [9], [10], [11].

Other mutations that may be behind “false OBI” are those frequently found in preS1/pre S2 regions. Mutations in these surface promoters are associated with reduced expression of HBsAg. As a result of these mutations, levels of HBsAg decrease significantly, or may even become undetectable [12].

Finally, splicing steps have a critical effect on gene expression in HBV. Guanine-to-adenine substitution at position 458 of the S gene interferes with the splicing of S gene messenger RNA and has been associated with the loss of HBAgs expression and low HBV DNA replication [13].

## Course of HBV infection and reactivation

Transmission routes of HBV are perinatal, parenteral, sexual, and through direct person-to-person contact [14]. In acute infection, the immune system activates and eliminates the hepatocytes infected. In 90% of neonates and 30% of children, who present an immune tolerant phase, progression to chronic disease is very frequent in the absence of adequate immunoprophylaxis [14, 15]. On the other hand, infection in young people and adults triggers a strong immune response.

Clinical resolution depends on interaction between immune response and virus replication activity. In most cases, when immunomodulatory mechanisms are effective, primo-infection resolves without specific symptoms. For this reason, HBV infection resolves spontaneously in above 95% of adults [14]. In 5–10% [15] of cases, excessive immunomodulation activates severe inflammatory response in the liver, causing symptoms of acute disease and a significant increase in aminotransferase concentrations, which generally resolves in less than 6 months. Exceptionally, in only 1% of cases, immune response may cause rapid massive lysis of the hepatocytes infected, thereby causing fulminant liver failure. Finally, in 5–10% of patients, the immune system is unable to control viral replication, and infection becomes chronic [15].

**Figure 2:**
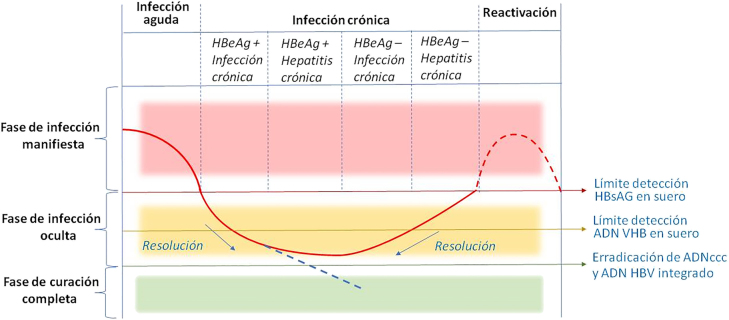
Phases of VHB persistence and reactivation. The continuous red curve indicates viral replication activity in acute, chronic, and occult phases. The blue dotted line indicates a hypothetic full resolution phase. The red dotted curve represents viral replication activity in the reactivation phase. HBeAg: hepatitis B e antigen; HBsAg: hepatitis B surface antigen; cccDNA: covalently closed circular DNA (modified from Shi et al. [16])

Chronic hepatitis B is a dynamic disease with different nonconsecutive phases in time. Figure 2 [16] summarizes the course of HBV infection, which is classified into four phases based on biochemical, serological and histological markers. These stages are classified based on HBeAg positivity, viral load (HBV DNA), ALT concentrations and grade of liver fibrosis (Table 2). Serial monitoring of these analytes is necessary to correctly stage patients. In 2017, the EASL (*European Association for the Study of the Liver*) defined the different phases with a new nomenclature, as detailed below [17]:

– **Phase 1: Chronic infection HBeAg-positive (phase of immunotolerance).** This phase is characterized by the presence of HBeAg in serum, very elevated levels of HBV DNA, and levels of ALT persistently within normal range. Liver inflammation and fibrosis are minimal or absent. However, the high level of HBV DNA suggests that hepatocarcinogenesis could be in progress in this early phase of infection. It is the most frequent phase in subjects infected perinatally and may extend for 10–30 years. These patients are highly contagious due to the high levels of HBV DNA.
– **Phase 2: HBeAg-positive chronic hepatitis B.** It is characterized by the presence of HBeAg, and high levels of HBV DNA and ALT. In the liver, there is moderate/severe neocroinflammation and accelerated progression of fibrosis. It is more frequent in subjects infected during adulthood, although it may also occur after several years of the immunotolerance phase. Most patients can achieve HBeAg seroconversion to anti-HBe and HBV DNA suppression and enter the HBeAg-negative infection phase.
– **Phase 3: HBeAg-negative chronic HBV infection, inactive carrier***.* It is characterized by the presence of serum antibodies to HBeAg, undetectable or low (<2,000 IU/mL) HBV DNA levels and normal ALT. In this phase, however, levels of HBV DNA may exceed 2,000 IU/mL (usually <20,000 UI/mL) with persistently normal levels of ALT with minimal liver inflammation and low grade of fibrosis. If this phase prolongs, patients have a low risk of progression to cirrhosis or hepatocellular carcinoma (HCC), although progression to chronic hepatitis may occur. Between 1 and 3% of patients may experience a spontaneous loss of HBsAg or seroconversion to anti-HBs. These patients generally present low levels of HBsAg in serum.
– **Phase 4: HBeAg-negative chronic hepatitis B.** It is characterized by the loss of HBeAg in serum, generally with detectable anti-HBe; fluctuating moderate or elevated levels of HBV DNA in serum (generally lower than in HBeAg-positive patients); and elevated ALT levels. Histological analysis of the liver demonstrates inflammation and fibrosis. This phase is associated with low rates of spontaneous remission of the disease.
– **Occult HBV infection.** As mentioned above, OBI is characterized by negative HBsAg with or without anti-HBc or anti-HBs antibodies. Patients exhibit normal levels of ALT and generally, but not always, undetectable levels of HBV DNA in serum. The liver shows detectable levels of covalently closed circular DNA (cccDNA). If the loss of HBsAg occurs before the onset of cirrhosis, it is associated with a minimal risk of cirrhosis, decompensation and HCC, and with higher survival rates. However, if the loss of HBsAg occurs in a patient with cirrhosis, monitoring is required due to the risk for HCC. Immunosuppression may cause HBV reactivation in these patients [17].

**Table 2:** Classification of chronic HBV infection phases.

HBV chronicity: HBsAg-positive (>6 months)				
	HBeAg-positive		HBeAg-negative	
	Phase 1. Chronic infection (phase of immunotolerance)	Phase 2. Chronic hepatitis	Phase 3. Chronic infection (inactive carrier)	Phase 4. Chronic hepatitis
HBV DNA	>10^7^ U/mL	10^4^–10^7^ U/mL	<2,000 UI/mL	>2,000 UI/mL
ALT	Normal	Elevated	Normal	Elevated
LF	No fibrosis/minimal	Moderate/severe	No fibrosis	Moderate/severe

ALT, alanine aminotransferase; LF, liver fibrosis.

## Molecular mechanism and immune response

The HBV genoma may not disappear completely after acute hepatitis, even after mild HBV infection. This is due to the fact that some HBV genomas remain as occult cccDNA in the liver. Their expression is largely controlled by the host immune system [18].

CccDNA is the mold of all viral transcripts and has the ability to generate infectious particles. HBV DNA may also integrate in the host genoma and remain in the hepatocyte of HBV-infected subjects, following spontaneous or treatment-induced HBsAg clearance. However, integrated HBV DNA is not replication-competent, does not generate virions, but may produce HBsAg as subviral particles through an independent pathway [16].

The molecular base of OBI is related to the stability and long-term persistence of cccDNA in the nucleus of infected hepatocytes. Epysomal cccDNA exists in the form of a viral minichromosome, similar to a plasmid, which is very stable and long lasting [16]. This added to the long half-life of the hepatocyte implies that HBV infection may continue for life [3]. Most cases of OBI present low levels of cccDNA in the liver. The suppression of replication activity and viral protein expression is modulated by the host immune system and epigenetic mechanisms. The low level of cccDNA with active transcription produces a low or undetectable transcription of RNA, thereby resulting in low protein translation and expression.

The high prevalence of OBI worldwide suggests that the immune system is effective in controlling HBV, although it does not disappear completely, as cccDNA persists for years.

Antiviral immune response is continuously stimulated via low intermittent concentrations of HBV antigens [3]. A small number of OBI cases originate from infection with HBV mutants with defective replication activity due to mutations in the S promoter genomic region. OBI shows long-lasting specific T-cell immune-response against HBV epitopes, with a more efficient response among seropositive OBI individuals [1].

## Diagnosis

Diagnosis of OBI is based on the detection of HBV DNA in blood or in the liver of HBsAg-negative individuals. The gold-standard method is the detection of HBV DNA in the liver; however, as it is a highly-invasive method, HBV DNA detection is performed in blood. Anti-HBc detection is often used instead of HBV DNA detection. Of note, these antibodies are negative in seronegative OBI. Therefore, diagnosis of OBI is established based on the detection of HBV DNA and HBsAg. HBsAg detection assays with inadequate sensitivity for the detection of HBV S variants (escape variants), may cause false negatives for HBsAg and lead to a misdiagnosis of OBI in subjects with evident HBV infection. Additionally, HBV DNA detection assays with inadequate sensitivity may yield false negatives and lead to undiagnosed OBI.

The limit of detection of HBsAg in commercially-available assays is 50 mIU/mL. Recent studies have developed antigen detection kits with a high sensitivity (10 times higher), known as ultrasensitive assays, such as “Lumipulse G HBsAg-HQ”, a chemiluminescent enzyme immunoassay, with a limit of detection of 5 mIU/mL [19, 20]. Other studies have demonstrated that the ultra-sensitive Abbot ARCHITECT assay, with a limit of detection of 5.2 mUI/mL for HBsAg, had a clinical performance near that of HBV DNA detection [21].

Other assays with higher sensitivity for the detection and determination of HBsAg have been recently developed, with a limit of detection of 0.5 mIU/mL. These assays could be used in patients with self-limited HBV to identify cccDNA transcriptional activity and minimal HBV replication, to detect OBI. Shinkai et al. conducted a comparative study of three HBsAg detection assays with different limits of detection (50.5 and 0.5 mIU/mL) in samples of serum of 120 patients with hematologic diseases who were receiving systemic chemotherapy. In 13 cases, HBV reactivation was detected through HBV DNA elevation. The authors suggest that the semi-automated immunoassay system combining CLEIA with the immune complex transfer method (ICT-CLEIA, Sysmex Corporation) has sensitivity for HBsAg similar to HBV DNA quantification. The ICT-CLEIA trial detected the presence of HBsAg in 12 of the 13 patients who presented reactivation, and even before HBV DNA elevation occurred in two cases Therefore, the ICT-CLEIA assay emerges as a new ultrasensitive HBsAg assay for monitoring HBV, avoiding HBV reactivation and preventing OBI transmission [22].

In addition to their high sensitivity, HBsAg reagents have different capacity to detect escape variants in the S region. The identification of variants of the S gene is essential for a correct diagnosis and its potential clinical implications. Therefore, for an optimal detection of these variants, the use of multivalent anti-HBs antibodies in HBsAg assays is recommended. The use of anti-HBs antibodies with multiple HBsAg epitopes in reagents should be required to guarantee correct HBsAg detection.

As mentioned above, the most efficient method for the diagnosis of OBI is the detection of HBV DNA replication in the liver. However, standard assays have not yet been validated for determination of HBV DNA replication. The detection of anti-HBc in blood can be a used as a substitute marker in the identification of OBI in patients who received immunosuppressive therapy and in epidemiologic studies. The Taormina group recommends their use when HBV DNA determination is not available. Currently anti-HBc detection assays have a high specificity (≥99%) [3].

## Prevalence of OBI

The prevalence of OBI worldwide has a large variability. In endemic regions such as Asia, prevalence may be as high as 8% [2, 6].

Reported prevalence ranged between 1 and 87%, although results should be interpreted with caution [2]. Hence, risk factors in the population of study, sampling problems, sensitivity of the assay used, and the prevalence of HBsAg in the geographic region of the study should be considered when estimating prevalence [3].

The prevalence of OBI is higher in subjects with risk factors for HBV infection i.e. patients with hepatitis C virus (HCV) co-infection (52%) [23] or human immunodeficiency virus (HIV) (15%) [24], patients on hemodialysis (2%) [25], or with cryptogenic cirrhosis (14%) [26].

The performance and sensitivity of HBV DNA and HBsAg detection, the characteristics of the population of study, the prevalence of infection with positive HBsAg in the general population, and the criteria used in the definition of OBI may influence the prevalence of OBI. Therefore, variability hinders comparative studies, and the prevalence in the general population remains unclear [3].

## Risk factors

The detection of risk factors for OBI is essential for the prevention of transmission. The main risk factors are shown in Table 3 [13].

**Table 3:** Risk factors for OBI.

Risk factors for OBI
Patients with previous history of HBV infections
Patients with HCV/HIV co-infections.
Organ transplant recipients
Blood donors
Organ donors
Patients with thalassemia or hemophilia
Patients with cryptogenic hepatitis, cirrhosis, and HCC
Patients on hemodialysis
Patient treated with lamivudine or interferon
Children of vaccination age, especially in regions where HBV is endemic
Immunocompromised patients due to biological treatments or chemotherapy (associated with anti-CD20 therapy)

## Patients with HCV co-infection

Current or previous HBV/HCV co-infection is relatively frequent, given that HBV and HCV share the same routes of transmission. In addition, OBI is frequently found in patients with chronic HCV infection.

According to recent studies, the prevalence of OBI among HCV patients is variable, ranging from 0 to 52% [23].

Mandour et al. conducted a study in the Suez Canal region of northeastern Egypt to determine the prevalence of OBI in patients with end-stage chronic renal insufficiency on hemodialysis, and in patients with chronic HCV. The prevalence of OBI was higher among HCV patients, as compared to hemodialysis patients, with prevalence rates ranging between 8.5 and 1.8%, respectively [27]. Emara et al. conducted another study in Egypt in 155 patients with chronic HCV receiving treatment with PEGylated interferon/ribavirin. The prevalence of OBI was estimated to reach 3.9% and affected younger patients, which is associated with a higher baseline hepatitis C viral load and lower liver fibrosis than in patients with monoinfection [28]. Bal et Onlen estimated that the prevalence of OBI in patients with chronic HCV infection treated with interferon was 1% [29].

The prevalence of OBI in patients with HCV co-infection can be due to several causes, including the mutations observed in the HBsAg gene in these patients [30]. Another cause could be low HBV replication, since the concurrent presence of the HBV and HCV genome in the same hepatocyte inhibits HBV replication due to the interference of HCV molecules [31].

The clinical impact of OBI in patients with chronic HCV is still unknown. Some studies suggest that the presence of OBI could be associated with more severe liver damage, cirrhosis and a higher rate of HCC. In some studies, OBI is also regarded as a cause of interferon therapy failure [32]. In contrast, other studies do not reveal any significant association between OBI and poor response to interferon/ribavirina [28]. The use of direct acting antivirals (DAAs) may improve HBV DNA detection in OBI patients, although it is a rare event without clinical or virological effects, at least in immunocompetent patients [33, 34].

To improve therapies for and effects of OBI, screening for anti-HBc antibodies and HBV DNA is recommended prior to initiation of treatment for HCV [11]. Treatment with DAAs has been associated with a higher risk for HBV reactivation in HBsAg-positive patients [35] and in some cases with severe hepatitis. HBV management guidelines recommend prophylaxis with nucleotide analogs in these patients during treatment. HBV reactivation in OBI may occur, with small elevations of HBV DNA without any clinical impact. Anyway, it is recommended to screen for HBsAg and anti-HBc in patients who are starting HCV therapy with DAA.

## Patients with HIV co-infection

OBI-HIV co-infection generally occurs in parenteral drug users, since HBV and HIV share the same route of transmission, as it occurs with chronic HCV infection.

The prevalence of OBI among HIV patients is uncertain, ranging from 0% [36] to 15% [24], with the clinical effects of co-infection been scarcely known. A retrospective study by Marquet–Juillet in 31 patients with HIV revealed the presence of HBV DNA in seven patients (22%). In this study, OBI was more frequent in patients with HCV co-infection. All presented anti-HBc antibodies. CD4+ cell count was significantly lower in the samples with detectable HBV DNA. The prevalence of OBI among patients with HIV infection and anti-HBc carriers was high, with a low hepatitis B viral load (<20 UI/mL) [37].

In Africa, especially in Sub-Saharian Africa, where HIV and HBV are prevailingly sexually transmitted, the prevalence of OBI was also variable, with prevalence rates of 5.3–18.7% in Kenya [38, 39]; 5.9% in Camerun [40]; 6.7 and 23% in South Africa [41, 42]; 15.1% in Sudan [43]; and 26.5% in Bostwana [44]. There is also a high variability across regions of the same country, as it occurs in Kenya and South Africa.

HIV infection exacerbates the course of chronic HBV infection, and may accelerate progression to liver fibrosis, cirrhosis and HCC. Patients with HBV/HIV co-infection show lower rates of spontaneous HBsAg and HBeAg seroconversion and a higher risk for HBV reactivation in inactive carriers. On the other hand, HBV does not influence the course of HIV disease [45]. Long lasting HBV and HIV may induce severe or fulminant hepatitis. For these reasons, screening for anti-HBc and HBV DNA is recommended prior to initiation of treatment in patients with HIV [13].

## Blood donors

The prevalence of OBI in blood donors is very low. In Europe, OBI has been identified in 1:1,000 to 1:50,000 blood donations [46]. According to the latest data available, it is estimated that the risk of OBI in our country is 1:170,000 [47]. In a study of blood donors in regions where HBV is highly endemic, the prevalence of OBI was very low, 0.11 and 0.13%, which translates into a very low impact on transfusion services [2]. In contrast, the incidence of HBV transmission via transfusion from OBI donors could be underestimated.

Several factors could explain this phenomenon: firstly, in most cases, HBV infection courses without clinical evidence of acute hepatitis in receptors. Secondly, levels of HBV DNA are extremely low or only detectable intermittently. Finally, the limited volume of donor archive samples may not allow HBV DNA determination [3].

Nucleic acid test (NAT) has higher sensitivity for the detection of HBV DNA than HBsAg assay, as a preventive measure against HBV transmission through blood transfusion [13]. The combination of the two tests successfully prevents most HBV transmissions. However, a residual risk may persist associated with extremely low HBV DNA levels and intermittent viral load in the blood of OBI donors. A range of studies reports that HBV transmission via transfusion of blood components of OBI donors contained a low viral load (<200 virions). Models based on clinical and experimental evidences estimate a residual risk of transmission of 3–14%, associated with transfusions from OBI donors with negative HBsAg and HBV loads [48]. Anti-HBc determination could improve the safety of blood donations [49]. In addition, the presence of anti-HBs in receptors reduces the risk for infection significantly [46].

In Spain, the Scientific Committee for Transfusional Safety (CCST) estimated that residual risk for transmission was higher in HBV than in HIV and HCV. In 2009, two options were suggested to reduce transmission; screening for anti-HBc vs. screening for HBV DNA in all donations. The former does not detect antibodies at onset of infection (due to the elevated prevalence and low specificity) and involves losing 4–5% of donations, which makes this option unfeasible. The second method detects infection since onset, and has the advantage of detecting OBI infections with undetectable HBsAg. Therefore, the CCST recommends routine screening for HBV via HBV DNA determination by NAT in all donations. In addition, the CST indicates that HBV DNA screening cannot be considered a substitute of HBsAg screening [47].

## OBI in patients on hemodyalisis

Patients on hemodialysis are at a higher risk of HBV infections, as they frequently receive transfusions, are immunocompromised, and undergo invasive procedures involving blood handling. Therefore, serological tests should be performed. In addition, all seronegative patients should receive immunization. In the last years, the prevalence of HBV in hemodialysis units has decreased significantly to 1.03% [50].

Diagnosis of liver damage based on levels of aminotransferases is challenging, since uremia may inhibit inflammatory reactions in the liver, thereby attenuating hepatocyte destruction.

A study conducted by Sowole et al. in a multiethnic cohort of patients on hemodialysis in London, with positive anti-HBc and negative HBsAg, revealed a prevalence of OBI of 2.2%. All patients exhibited very low viral loads (<10 IU/mL), with a low risk for nosocomial transmission, due to the presence of a robust active immunization plan against HBV. A limitation of this study was that viral load was only determined once, which does not exclude higher viral loads in these patients, as viral load may be intermittent [25].

HBV DNA quantification is the most efficient method for detecting OBI in patients on hemodialysis. Therefore, routine screening for HBV and OBI, among other infections, is recommended in patients on hemodialysis, using techniques with a high molecular sensitivity to prevent nosocomial transmission.

## OBI and cryptogenic liver disease

Cryptogenic liver disease is characterized by an unknown etiology, and prevalence varies largely across the world. OBI has been demonstrated in patients with persistently elevated ALT concentrations of unknown origin, and has been considered an additional risk factor for progression to cirrhosis and HCC. Hashemi et al. assessed the prevalence of OBI in a cohort of 50 patients with crytogenic cirrhosis and 80 healthy controls. A total of 7(14%) patients in the group of patients with cryptogenic cirrhosis were positive for HBV DNA determined by PCR. Four of these patients (57%) were seronegative, i.e. they had negative anti-HBc and anti-HBs markers. In the control group, there were not patients positive for HBV DNA. The authors concluded that OBI is frequent in patients with cryptogenic cirrhosis, especially in older patients. This may contribute to rapid progression to liver decompensation, which warrants further studies on OBI in patients with liver cirrhosis [26].

To improve the treatment and management of patients with cryptogenic liver disease, HBV DNA determination by high-sensitive molecular assays is recommended before the patient develops signs of cirrhosis or HCC [13].

## Conclusions

OBI may persist life long after infection, due to the high life of the HBV genoma, which includes cccDNA and DNA integrated in the host genoma.

A high number of patients with OBI may remain undiagnosed.

Detecting risk factors is essential to prevent the two main problems associated with OBI: HBV reactivation and transmission.

Screening for OBI via HBV DNA quantification in patients with risk factors is essential. HBV serology including HBsAg, anti-HBc and anti-HBs determination should also be performed.

There are some clinical and biological aspects of OBI that need further research. Finally, it is necessary that validated, standard, ultra-sensitive HBsAg assays are developed to detect S variants in serum that are sensitive to HBsAg fragments, added to other assays for detecting cccDNA and other forms of HBV viral genoma in the liver.
